# Somatosensory omissions reveal action‐related predictive processing

**DOI:** 10.1002/hbm.26550

**Published:** 2023-12-05

**Authors:** Tjerk T. Dercksen, Andreas Widmann, Tömme Noesselt, Nicole Wetzel

**Affiliations:** ^1^ Research Group Neurocognitive Development Leibniz Institute for Neurobiology Magdeburg Germany; ^2^ Center for Behavioral Brain Sciences Magdeburg Germany; ^3^ Wilhelm Wundt Institute for Psychology Leipzig University Leipzig Germany; ^4^ Department of Biological Psychology Otto‐von‐Guericke‐University Magdeburg Magdeburg Germany; ^5^ University of Applied Sciences Magdeburg‐Stendal Stendal Germany

**Keywords:** EEG, efference copy, motor, predictive coding

## Abstract

The intricate relation between action and somatosensory perception has been studied extensively in the past decades. Generally, a forward model is thought to predict the somatosensory consequences of an action. These models propose that when an action is reliably coupled to a tactile stimulus, unexpected absence of the stimulus should elicit prediction error. Although such omission responses have been demonstrated in the auditory modality, it remains unknown whether this mechanism generalizes across modalities. This study therefore aimed to record action‐induced somatosensory omission responses using EEG in humans. Self‐paced button presses were coupled to somatosensory stimuli in 88% of trials, allowing a prediction, or in 50% of trials, not allowing a prediction. In the 88% condition, stimulus omission resulted in a neural response consisting of multiple components, as revealed by temporal principal component analysis. The oN1 response suggests similar sensory sources as stimulus‐evoked activity, but an origin outside primary cortex. Subsequent oN2 and oP3 responses, as previously observed in the auditory domain, likely reflect modality‐unspecific higher order processes. Together, findings straightforwardly demonstrate somatosensory predictions during action and provide evidence for a partially amodal mechanism of prediction error generation.

## Graphical abstract

Participants repeatedly pressed a button that mostly (88%), sporadically (50%), or never (motor) resulted in a tactile stimulus. Comparing tactile omissions between conditions, a brain response was only recorded in the 88%‐condition, where a stimulus prediction was present. This response presumably reflects prediction error on multiple levels in the brain.

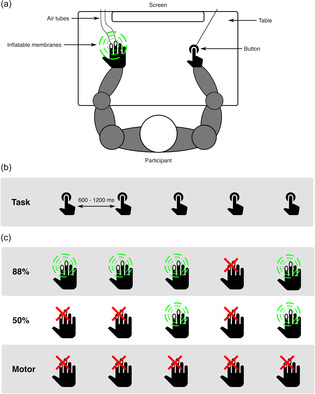

## 1 INTRODUCTION

Whether mindlessly playing with our pen, or consciously tapping on our phone, it seldomly happens that we are surprised by the tactile sensations that our own actions produce. Several models explain this phenomenon in terms of an action‐related sensory prediction that attenuates surprise. For example, motor commands are thought to be accompanied by an efference copy that signals the predicted sensory consequences of the action (Sperry, 1950; von Holst & Mittelstaedt, 1950). Similarly, predictive coding assumes a cortical hierarchy where higher cortical levels predict lower levels (Friston, 2005; Rao & Ballard, 1999). As action unfolds, motor areas are thought to send predictions to sensory areas, where they are compared to actual input (Adams et al., 2013; Friston et al., 2017). Where predictions are incorrect, a prediction error is propagated back up the hierarchy that corrects higher‐level models, while correct predictions result in diminished prediction error or surprise compared to external stimuli.

Efference copy and predictive coding, along with comparable forward models, explain a variety of behavioural (e.g., Bays et al., 2005, 2006; Kilteni & Ehrsson, 2017a, 2017b; Walsh et al., 2011) and neuroimaging findings of perceptual phenomena (De Lange et al., 2018; Horváth, 2015; Imamizu, 2010; Schröger et al., 2015; Shadmehr et al., 2010; Shin et al., 2010). For example, sensory attenuation or suppression has consistently been reported in several modalities, with diminished neural activity for self‐generated versus externally generated stimuli (Bäß et al., 2008; Bednark et al., 2015; Blakemore et al., 1998, 1999, 2000; Kilteni & Ehrsson, 2020; Knolle et al., 2013b, 2019; Roussel et al., 2013, 2014; Shergill et al., 2013). However, the observed attenuation in these studies is only indirect evidence of a hypothetical sensory prediction, leaving room for explanations other than prediction‐related effects such as neural adaptation (Schröger et al., 2015). A more explicit demonstration of motor‐induced sensory predictions is found in studies using auditory stimulus omissions. Here, an action is reliably coupled to a sound that is sometimes unexpectedly omitted. An increasing number of studies have demonstrated omission‐related brain responses when auditory stimuli were coupled to an action (Dercksen et al., 2020, 2022; Korka et al., 2020; SanMiguel, Saupe, & Schröger, 2013; SanMiguel, Widmann, et al., 2013) or another stimulus (Stekelenburg & Vroomen, 2015; van Laarhoven et al., 2017). Event‐related potentials (ERPs) in these studies show a consistent pattern of omission responses: an initial oN1 component (~100 ms) potentially reflecting sensory prediction error, followed by a later oN2 and possibly several oP3 components likely reflecting higher‐level processing. Explaining this sensory‐related neural activity in the absence of a stimulus is not possible without the notion of an internal process that triggers this activity, which is often interpreted in terms of prediction and prediction error.

Stimulus omission paradigms avoid confounding bottom‐up activity caused by unexpected deviant stimuli and are thus well suited to investigate motor‐induced sensory predictions (Heilbron & Chait, 2018; Korka et al., 2021; Schröger et al., 2015). Moreover, the cascade of subsequent omission components as observed in auditory studies offers a detailed insight into the subprocesses related to prediction error computation. Despite these advantages, omission studies are still scarce compared to other paradigms investigating predictions and the omission response has barely been studied outside the auditory modality. Three studies have demonstrated somatosensory omission responses, all using MEG (Andersen & Dalal, 2021; Andersen & Lundqvist, 2019; Tesche & Karhu, 2000). These studies did not involve action but used a fixed interstimulus interval to induce stimulus predictions, all reporting only a single omission‐related component. Possibly, the lack of a time‐locking cue might have led to decreased power hindering the observation of all components of the omission response.

In the current study, we therefore aimed to characterize the full omission‐related response. To this end, we recorded action‐induced somatosensory omission responses using ERPs. The paradigm was similar to aforementioned auditory studies. A two‐step approach was used to analyse ERPs, starting with cluster‐based permutation tests to determine significant variation of the signal between conditions and following up with temporal principal component analysis (PCA) for a more detailed examination of the effects. PCA, as compared to conventional ERP analysis, mitigates the problem that the observed peaks of the recorded ERP waveform are a poor indication of its underlying components (Scharf et al., 2022). It achieves this by decomposing the waveform into components using a factor analytic approach. An added advantage of applying PCA in the current study is that it facilitates a comparison of omission responses across modalities, as this method was also used in previous auditory omission studies (Dercksen et al., 2020, 2022; Korka et al., 2020).

## 2 MATERIALS AND METHODS

### 2.1 Participants

EEG and behavioural data were acquired from a total of 30 participants (17 females; age range = 19–39; mean age = 25 years, *SD* = 5 years; one left‐handed as measured by an adapted German version of the Oldfield Scale; Oldfield, 1971; the left‐handed participant performed the task with the same hand as did right‐handed participants). All participants were compensated either financially or in the form of credit points. Participants gave written consent prior to the experiment. The project was approved by the local ethical committee.

### 2.2 Apparatus and stimuli

Participants were seated in a dimly lit, electrically shielded and acoustically attenuated chamber, while EEG was continuously recorded. The experiment was programmed using Psychtoolbox (version 3.0.15; Brainard & Vision, 1997) and ran on a Linux‐based system using GNU Octave (version 4.0.0). A white fixation cross was presented using a VIEWPixx/EEG Display (Resolution 1920(H) × 1080(V)—23.6‐in. display size). The fixation cross was presented in the middle of a grey screen, at about 60 cm from the participants' eyes (0.67° × 0.67° visual angle). To trigger the stimuli (or omissions), a custom‐built button was used in order to ensure a completely silent button press. The button used an infrared photoelectric mechanism and was additionally padded with sound absorbing material. To ensure that no residual sound (e.g., contact of the skin of the fingertip with the button surface) was correlated with the button press and membrane inflation, participants wore Sennheiser HD‐25 headphones during the experiment (no sound was presented). Tactile stimuli were presented using a pulse of pressurized air (3 bar) that inflated a membrane, which was controlled using a somatosensory stimulus generator (University of Münster, Germany) that was placed outside the chamber. Stimulus duration was approximately 30 ms. Two membranes were placed on the left middle and index fingers at the volar aspect of the distal phalanx. The stimulation of two fingers was chosen because this generates a stronger signal compared to one finger (Severens et al., 2010) but at the same time is still focused to a limited part of the cortex. The tactile stimulus always consisted of simultaneous stimulation of both fingers. Because of the travel time of the air pulse, there was a slight time delay between button press and inflation of the membrane (onset of the tactile stimulus) of approximately 40 ms (which was corrected during data preprocessing of the ERPs, see Section 2.5). The delay varied over a range of max. 4 ms.

### 2.3 Experimental design

**FIGURE 1:**
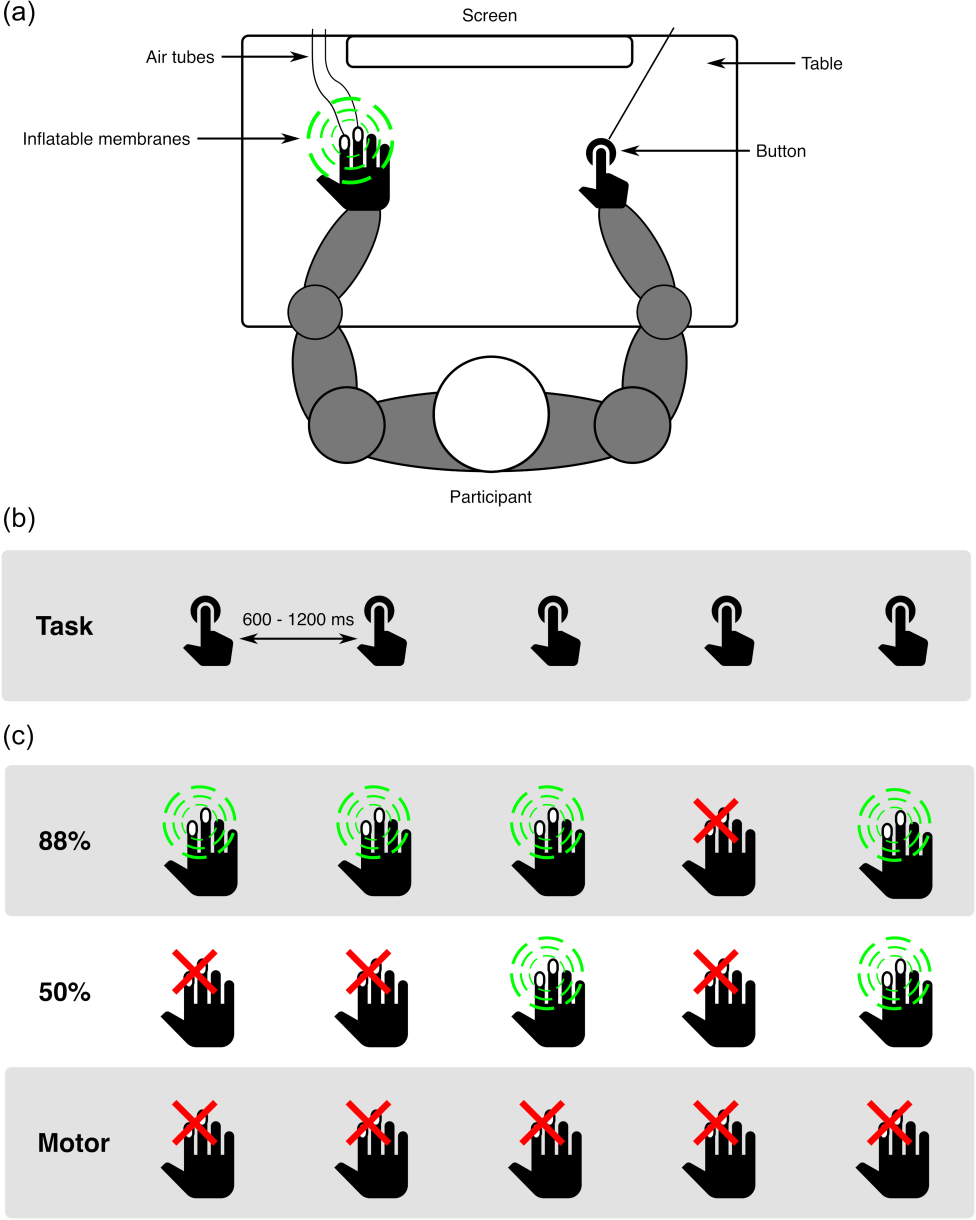
Schematic representation of the experimental design. Panel (a) shows the experimental set‐up: a participant sat in front of a screen with both arms on a table. With the right hand a button was pressed, possibly resulting in a stimulus on the left hand (indicated with green circles). Stimuli were applied by a puff of air traveling through air tubes and inflating a membrane on the left middle‐ and index‐finger. Panel (b) depicts the task over time, where participants pressed a button every 600–1200 ms. Panel (c) shows examples of the tactile effects of the button presses for all three conditions. In the 88%‐condition, there was an 88% chance of a button press resulting in a stimulus. In the 50%‐condition, the chance was 50%. In the motor condition, button presses never resulted in a tactile stimulus.

The experimental task was adapted from an auditory omission study by SanMiguel, Widmann, et al. (2013). Participants sat approximately 60 cm from a screen, having their right index finger on a button, and their left hand (where the tactile stimulus was applied) on a table. Distance between hands was approximately shoulder width (see Figure 1a for experimental layout). In all conditions, participants were asked to press a button every 600–1200 ms while looking at the fixation cross (Figure 1b). Two distinct tactile conditions (88%‐condition, 50%‐condition) and a motor control condition were presented (Figure 1c). In the tactile conditions, a button press resulted in a tactile stimulus either 88% (88%‐condition) or 50% (50%‐condition) of the time. In the remaining percentage of the button presses, the tactile stimulus was omitted. In the motor control condition only the button was pressed, never resulting in a tactile stimulus. This condition was included to be able to subtract the neural activity related to the pressing of the button. A total of 160 omissions and 1120 tactile stimuli were presented in the 88%‐condition, a total of 160 omission and 160 tactile stimuli were presented in the 50%‐condition, and a total of 320 trials were presented in the motor control block. Blocks in the 88%‐condition consisted of 20 omissions and 140 tactile stimuli, blocks in the 50%‐condition consisted of 80 omissions and 80 tactile stimuli, and motor control blocks consisted of 160 trials. In the 88%‐condition, omissions were randomly placed, under the restricting conditions that the first five trials of every block were always tactile trials, and every two trials following an omission were always tactile trials. In the 50%‐condition, omission and tactile trials were randomly mixed. Before the experiment, two short training blocks (60 trials each block) were completed where participants trained to press the button every 600–1200 ms. In these training blocks, feedback was presented visually after every button press, displaying the number of milliseconds that was in between the last button presses. In the first training block, no tactile stimuli were presented when pressing the button, while in the second training block a tactile stimulus was always presented when pressing the button. After this training, 12 experimental blocks were presented. Block order was identical for all participants, first presenting a motor control block, followed by eight blocks of the 88%‐condition, then two blocks of the 50%‐condition, and ending with another motor control block. The order of blocks was chosen with possible transfer effects in mind (SanMiguel, Widmann, et al., 2013). The 50%‐block could have induced a learning effect that there is no reliable coupling between button‐press and stimulus, possibly resulting in absent omission responses if the 88%‐blocks were presented after the 50%‐blocks. Therefore, it was decided to always present 50%‐blocks after 88%‐blocks, since learning effects from 88% to 50%‐blocks would be less problematic. If learning effects would be present, resulting in a significant omission result in the 50% condition, more participants would have been measured where the block order would be reversed (50%‐condition before 88%‐condition). However, this was not necessary as no significant omission responses were measured in the 50%‐condition after 30 subjects. Total experiment time was about 45 min including breaks.

### 2.4 Data recording

EEG was recorded from a total of 63 active electrodes, placed according to the extended international 10‐10 system at the following positions: Fp1, Fz, F3, F7, FC5, FC1, C3, T7, CP5, CP1, Pz, P3, P7, O1, O2, P4, P8, CP6, CP2, Cz, C4, T8, FC6, FC2, F4, F8, Fp2, AF7, AF3, AFz, F1, F5, FT7, FC3, C1, C5, TP7, CP3, P1, P5, PO7, PO3, POz, PO4, PO8, P6, P2, CPz, CP4, TP8, C6, C2, FC4, FT8, F6, AF8, AF4, F2, and the left (M1) and right (M2) mastoids. Furthermore, EOG was recorded from three electrodes placed left and right of the outer canthi of the eyes and below the left eye. The reference electrode was placed on the tip of the nose, and a ground electrode was placed at position Fpz. An Actichamp amplifier (BrainProducts, Gilching, Germany) was used, recording at 500 Hz, DC‐coupled and with a 140 Hz low‐pass filter using BrainVision Recorder software (version 1.21). Data are available on request to the corresponding author without further conditions.

### 2.5 EEG data preprocessing

EEG data analysis was performed with MATLAB software using the EEGLAB toolbox (Delorme & Makeig, 2004). Timestamps of the triggers were corrected for the delay between button press and stimulus by adding 40 ms to each timestamp. Data were filtered offline with a 0.1 Hz high‐pass filter (−6 dB, Kaiser windowed sinc FIR filter, order = 8024, beta = 5, transition bandwidth = 0.2 Hz) and a 48 Hz low‐pass filter (−6 dB, Kaiser windowed sinc FIR filter, order = 402, beta = 5, transition bandwidth = 4 Hz, this low‐pass filter has full attenuation at 50 Hz power line frequency). Data were segmented into epochs starting 200 ms before and ending 500 ms after (corrected) stimulus/omission onset. When a trial was pressed either less than 600 ms or more than 2000 ms after the preceding trial, the trial was excluded. Although subjects aimed to press between 600 and 1200 ms, there was no reason to discard trials pressed slightly later. Only a 2000 ms cut‐off was applied to eliminate trials where subjects would forget to press the button. Based on this criteria, on average 14 trials were rejected per participant (median = 1, min/max = 0/218, *SD* = 46). Noisy channels were removed from the data, which were defined as having a robust z‐score of the robust standard deviation (0.7413 times the interquartile range) larger than 3 (Bigdely‐Shamlo et al., 2015). These channels were removed from analysis and interpolated after independent component analysis (ICA). Epochs exceeding a 500 μV signal‐change per epoch threshold were removed. Based on this criteria, on average 17 trials were rejected per participant (median = 5, min/max = 0/116, *SD* = 29). ICA was performed to correct for artefacts. This was done on data which were 1 Hz high‐pass filtered (−6 dB, Kaiser, order = 1604, beta = 5, transition bandwidth = 1 Hz) and 48 Hz low‐pass filtered (same as above), as 1–2 Hz high‐pass filters improve ICA performance (Klug & Gramann, 2021; Winkler et al., 2015). Epoching and channel and trial removal were identical to the 0.1 Hz filtered dataset. After ICA, the obtained demixing matrix was subsequently applied to the 0.1–48 Hz filtered data. Two independent raters judged components, aiming to remove all heart‐, eye‐, and muscle‐related components. Raters specifically paid attention to not remove components that indicated neural activity, considering the frequency spectrum (in particular alpha peak; Chaumon et al., 2015; Winkler et al., 2011), topography and event‐related average of the components. Selected components were then discussed to come to a final judgement of components to be removed. Artefact independent components (ICs) were detected with support of the IClabel plugin (Pion‐Tonachini et al., 2019). On average, 15 components were rejected per participant (median = 15, min/max = 11/21, *SD* = 4). Each epoch was baseline corrected by subtracting the mean amplitude of the −200 to −100 ms window preceding stimulus onset (corrected for delay between button press and stimulus). Although this window might include motor‐related activity (e.g., planning, execution), this should be common to all conditions. To further ensure that our baseline approach did not drive the omission effects observed in this study, we performed *t*‐tests equivalent to those reported in Section 3.3 on non‐baselined data. These tests confirmed that the omission effects observed in Section 3.3 are still elicited using non‐baseline corrected data, excluding the possibility that they were driven by the baseline correction. The first five trials of each block and the two trials following an omission in the 88% condition were excluded from analysis to prevent confounding activity unrelated to the stimulus (e.g., attention‐related activity). Finally, trials that exceeded 125 μV signal‐change per epoch were excluded from analysis. Based on this criteria, on average, 26 trials were rejected per participant (median = 7, min/max = 0/405, *SD* = 73). In total, on average 1802 trials were left per participant after preprocessing (median = 1838, min/max = 1451/1856, *SD* = 87). The stimulus ERP in the 88%‐condition was based on an average of 738 trials per participant (median = 750, min/max = 600/759, *SD* = 37). The omission ERP in the 88%‐condition was based on an average of 156 trials per participant (median = 159, min/max = 126/160, *SD* = 8). The stimulus ERP in the 50%‐condition was based on an average of 150 trials per participant (median = 153, min/max = 102/158, *SD* = 12). The omission ERP in the 50%‐condition was based on an average of 148 trials per participant (median = 153, min/max = 88/157, *SD* = 14). The ERP in the motor condition was based on an average of 301 trials per participant (median = 306, min/max = 251/310, *SD* = 13). Condition‐specific ERPs were computed for each participant.

### 2.6 Behavioural data

Behavioural data were analysed to check for systematic differences between conditions regarding the temporal asynchrony between button presses. The asynchrony was determined on the basis of the behavioural data from which any too early/late button presses were removed. Trials were defined as too early when time between button presses was less than 600 ms, and as too late when time between button presses exceeded more than 2000 ms.

### 2.7 ERP analysis

Two distinct approaches were employed for ERP analysis. First, a cluster‐based permutation test was performed to demonstrate differences between the raw ERPs. Second, in order to provide a comprehensive assessment of these effects, a temporal PCA was performed.

#### 2.7.1 Cluster‐based permutation tests

Cluster‐based permutation tests were performed using the FieldTrip toolbox (Oostenveld et al., 2011) on a time‐window of −200 to 500 ms around the (corrected) stimulus/omission onset. Cluster‐based permutation tests employ a nonparametric statistical approach to assess differences between conditions, where observed clusters of adjacent data points are identified and compared to a randomly shuffled null distribution. This method effectively safeguards against type I errors in EEG data (Maris & Oostenveld, 2007), making it highly suitable to determine whether differences between conditions are present. Parameters were kept as suggested by the Fieldtrip tutorial on cluster‐based permutation tests, with temporo‐spatial clusters defined by a minimum of three neighbouring channels, using Monte Carlo method to calculate the *p*‐value, dependent samples *t* test as statistic, “cluster” as correction method, “maxsum” as cluster statistic, .025 as alpha, and 1000 randomizations. For omission responses, the following contrasts were tested: 88%‐condition versus motor‐control, 50%‐condition versus motor‐control, and 88%‐condition versus 50%‐condition. For somatosensory responses, only the 88%‐condition versus 50%‐condition contrast was tested in order to obtain insights regarding sensory attenuation. A cluster was considered statistically significant when the *p*‐value was below .05.

#### 2.7.2 Principal component analysis

Although cluster‐based permutation tests can reveal differences between conditions, more detailed inferences about latency and location (at sensor level) are unjustified (Sassenhagen & Draschkow, 2019). Therefore, we computed temporal PCA on the grand‐average ERP data (including one individual average waveform per participant, condition, and electrode) to analyse ERPs in greater detail. This method aims to statistically decompose ERP waveforms into the constituent components that constitute the resulting waveform (see Dien, 2012 or Scharf et al., 2022 for tutorial treatments). The number of retained components was determined using Horn's parallel test, which compares the variance explained by each factor with the variance explained by the corresponding factor from a simulated dataset of uncorrelated (noise) variables (Scharf et al., 2022). An R (R 4.1.2; R Core Team, 2021) implementation of the Geomin rotation (Yates, 1987) method with *ε* = 0.01 was applied to the initial PCA solution as described in the tutorial of Scharf et al. (2022). Geomin rotation is less prone to conflating components (representing separate components in a single factor) with strong temporal and spatial overlap than other rotation methods like Promax (Scharf & Nestler, 2018, 2019). Two separate PCAs were computed, one to analyse ERP responses to tactile omissions and one to analyse ERP responses to tactile stimuli. The motor‐control condition was included in both PCAs to control for the neural activity associated with the pressing of the button. Note that by including the motor‐control condition in both PCAs, there is a possibility of artificial similarities between the results of both PCAs. The PCA of omission responses (plus motor‐control) was computed on the individual averages of the motor control, 88%‐condition omissions, and 50%‐condition omissions together, resulting in identical components for all experimental conditions which could vary in amplitude across conditions. The PCA of tactile stimuli responses (plus motor‐control) was again computed on the individual averages of the motor control, 88%‐condition stimuli, and 50%‐condition stimuli together. From a theoretical perspective, only the stimulus responses in the time‐window of the initial sensory omission responses were of interest to this study. Components outside this time‐window were not considered for further analysis. As no prior information was available, statistical regions of interest for both PCAs were based on visual inspection of the topographies of the individual components. For early oN1 using right temporal electrodes: C6, CP6. For late oN1 using bilateral temporal electrodes: C4, C6, FT7. For oN2 using frontal electrodes: Fz, F2. For oN3 using occipital electrodes: P5, P6, P7, P8. For oP3‐1 using central electrode: Cz. For oP3‐2 using central electrode: Cz. For oP3‐3 using frontal electrode: FC2. For oP3‐4 using right temporal electrode: C4. For oP3‐5 using right temporal electrodes: C2, CP2, C4, CP4. For tactile components, we will refer to their order of occurrence in the PCA. For tactile component, eight using right temporal electrode: CP6. For tactile component, five using right temporal electrodes: C4, C6. For tactile component, two using central/parietal electrodes: Cz, CPz. Resulting PCA components are ordered by explained variance with the first component explaining most variance. Explained factor variance is computed as the ratio of variance accounted for by a factor (sum of the variance multiplied by the factors' loading matrix and correlation matrix) and the overall total variance (sum of the variance).

Although in this study, the analysis used for component separation is referred to as PCA; technically, the algorithm estimates an exploratory factor analysis (EFA). Because differences between PCA and EFA estimates are negligible (see Scharf et al., 2022, footnote 11) and the term PCA is dominant in the field, this article will keep referring to PCA with this technicality in mind.

### 2.8 Statistical analysis

Statistical testing was done using a Bayesian approach. Additionally, we report frequentist statistics. This way, readers familiar with Bayesian statistics can benefit from its advantages (Rouder et al., 2009; Wagenmakers, 2007), for example, direct interpretability and the evaluation of the evidence for the null model provided by the data, while still keeping our results interpretable for readers preferring frequentist statistics and allowing a simple comparison with frequentist results from previous publications.

Behavioural data were tested for differences between conditions regarding the time asynchrony between button presses. A one‐way repeated‐measures ANOVA was performed using condition (motor, 88%‐condition, 50%‐condition) as independent variable and mean asynchrony between button presses as dependent variable. Equivalent variables (condition, mean asynchrony between button presses) were used for the Bayesian repeated measures ANOVA. Follow‐ups were performed using paired samples *t* tests, corrected for multiple comparisons using Bonferroni correction (Bonferroni, 1936) correcting for a family of three (motor, 88%‐condition, 50%‐condition), as well as Bayesian paired samples *t* tests.

PCA omission and stimulus components were tested for differences between conditions using separate paired samples *t*‐tests (88%‐condition vs. motor control, 50%‐condition vs. motor control, 88%‐condition vs. 50%‐condition). Equivalent comparisons were tested using Bayesian paired samples *t* tests (88%‐condition vs. motor control, 50%‐condition vs. motor control, 88%‐condition vs. 50%‐condition).

All statistical tests were performed in JASP (version 0.16.0 JASP Team, 2021). For Bayesian statistics, the null hypothesis corresponded to a standardized effect size *δ* = 0, while the alternative hypothesis was defined as a Cauchy prior distribution centred around 0 with a scaling factor of *r* = .707 (the default “medium” effect size prior scaling). Additionally, for the Bayesian repeated measures ANOVA (see Rouder et al., 2017 for more information on Bayesian ANOVA), the JASP default fixed (condition) and random (participant variability) effects priors were used, defined as, respectively, *r* = .5 and *r* = 1. Resulting Bayes factors (*BF*_*10*_) were interpreted following Lee and Wagenmakers (2013), who give the labels anecdotal (0.33–3), moderate (3–10 or 0.33–0.1), strong (10–30 or 0.1–0.033), and very strong (>30 or <0.033) for specific ranges of the BF. We replaced the label “anecdotal” with “weak,” and “very strong” with “decisive” to aid interpretation.

## 3 RESULTS

**FIGURE 2:**
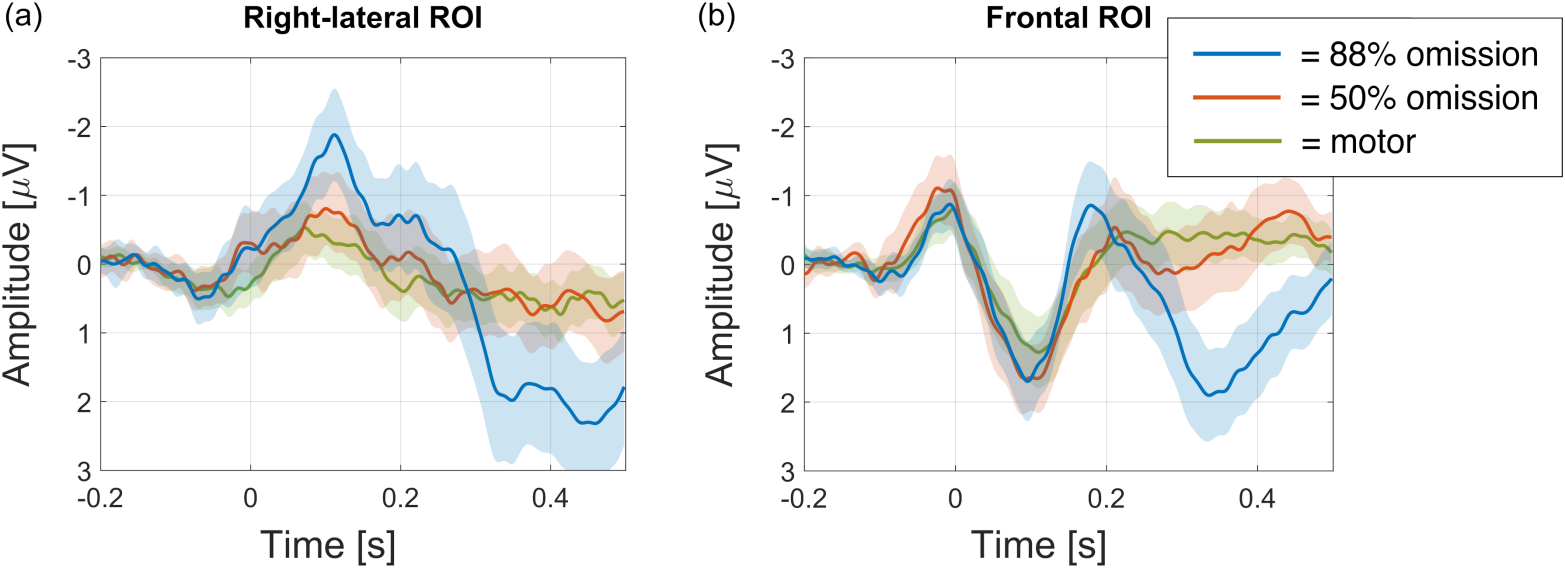
Uncorrected event‐related potentials (ERPs) for right‐lateral ROI (a: channels C6, CP6) and frontal ROI (b: channels Fz, F2). Plots show ERPs including 95% CIs for 88% omission, 50% omission, and motor‐control conditions.

This paradigm compared physically identical stimuli (a silent button press) between conditions that manipulate the prediction related to the button press. Assuming that the motor‐control condition does not predict a somatosensory stimulus on the left fingers, any additional activity in the other conditions (88%‐ and 50%‐conditions) was considered prediction‐related activity. Uncorrected ERP results are shown in Figure 2. As prediction‐related activity is the main focus of this study, Figures 4, 5, 6 show difference waves where the motor‐control condition was subtracted from the other conditions.

### 3.1 Behaviour

Participants were generally able to keep a stable pace between button presses throughout the experiment, where the aim was to keep inter‐press interval (IPI) between 600 and 1200 ms. Group average was 1001 ms (*SD* = 112 ms) for motor‐control, 965 ms (*SD* = 120 ms) for 88%‐condition, and 937 ms (*SD* = 122 ms) for 50%‐condition. Repeated measures ANOVAs showed decisive evidence for a difference between conditions (*BF*_10_ = 427, *F*_(2,58)_ = 11.862, *p* < .001, *η* ^2^ = 0.290). Post hoc *t* tests showed moderate evidence for longer IPI in motor versus 88%‐conditions (*BF*_10_ = 5.096, *d* = 0.50, *t*(29) = 2.760, *p*_*bonf*_ = .023), decisive evidence for longer IPI in motor versus 50%‐conditions (*BF*_10_ = 258, *d* = 0.89, *t*(29) = 4.856, *p*_*bonf*_ < .001), and weak evidence for longer IPI in 88%‐ versus 50%‐conditions (*BF*_10_ = 1.667, *d* = 0.38, *t*(29) = 2.095, *p*_*bonf*_ = .122). Although behavioural differences were observed between conditions, mean differences were small (maximal 64 ms) and therefore unlikely to systematically affect the motor‐related neural activity between conditions.

### 3.2 Cluster‐based permutation tests

**FIGURE 3:**
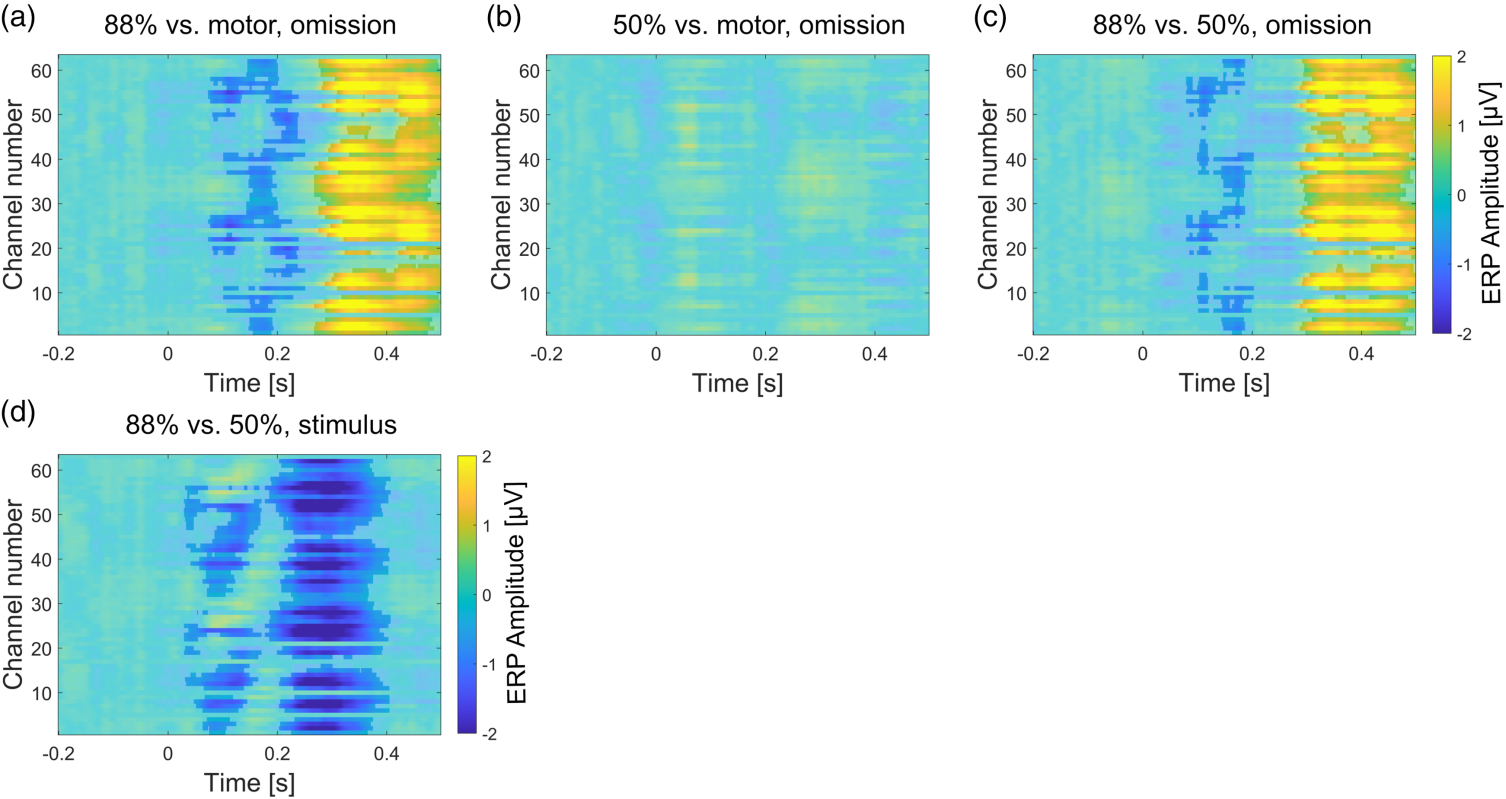
Event‐related potential (ERP) amplitudes (colour map) and cluster statistics (transparency maps) for the difference between 88%‐ minus motor‐condition contrast in omission trials (panel a), 50%‐ minus motor‐condition contrast in omission trials (panel b), 88%‐ minus 50%‐condition contrast in omission trials (panel c), and 88%‐ minus 50%‐condition contrast in stimulus trials (panel d). Colour maps display the difference in ERP amplitude over time, broken down by electrode. Electrode numbers, broadly, begin at left frontal sites, ascending counter clockwise, first to posterior sites and then to right frontal sites. Statistically significant clusters (*p* < .05) are shown as opaque, while nonsignificant sampling points are shown as transparent.

Cluster‐based permutation testing of the 88%‐condition (omission) vs. the motor‐control condition indicated an effect of condition, showing two significant clusters (Figure 3a). The range of the first cluster (*p* = .008) was around 80–250 ms and included electrodes: Fp1, Fz, F3, F7, FC5, FC1, C3, T7, CP5, P3, P7, O1, O2, P4, P8, CP6, CP2, C4, T8, FC6, FC2, F4, F8, Fp2, AF7, AF3, AFz, F1, F5, FT7, FC3, C5, TP7, CP3, P5, PO7, PO3, POz, PO4, PO8, P6, P2, CP4, TP8, C6, C2, FC4, FT8, F6, AF8, AF4, F2. The range of the second cluster (*p* < .001) was around 270–500 ms and included electrodes: Fp1, Fz, F3, F7, FC5, FC1, C3, T7, CP5, CP1, Pz, P3, P7, O1, O2, P4, P8, CP6, CP2, Cz, C4, T8, FC6, FC2, F4, F8, Fp2, AF7, AF3, AFz, F1, F5, FT7, FC3, C1, C5, TP7, CP3, P1, P5, PO7, PO3, POz, PO4, PO8, P6, P2, CPz, CP4, TP8, C6, C2, FC4, FT8, F6, AF8, AF4, F2.

Cluster‐based permutation testing of the 50%‐condition (omission) versus the motor‐control condition indicated no effect of condition.

Cluster‐based permutation testing of the 88%‐condition (omission) versus the 50%‐condition (omission) indicated an effect of condition, showing two significant clusters (Figure 3b). The range of the first cluster (*p* = .024) was around 80–200 ms and included electrodes: Fp1, Fz, F3, F7, FC5, FC1, C3, T7, CP5, CP1, Pz, P3, P4, P8, CP6, CP2, Cz, C4, T8, FC6, FC2, F4, F8, Fp2, AF7, AF3, AFz, F1, F5, FT7, FC3, C1, C5, TP7, CP3, P1, P5, POz, P6, P2, CPz, CP4, TP8, C6, C2, FC4, FT8, F6, AF8, AF4, F2. The range of the second cluster (*p* < .001) was around 290 and 500 ms and included electrodes: Fp1, Fz, F3, F7, FC5, FC1, C3, T7, CP5, CP1, Pz, P3, P7, O1, O2, P4, P8, CP6, CP2, Cz, C4, T8, FC6, FC2, F4, F8, Fp2, AF7, AF3, AFz, F1, F5, FT7, FC3, C1, C5, TP7, CP3, P1, P5, PO7, PO3, POz, PO4, PO8, P6, P2, CPz, CP4, TP8, C6, C2, FC4, FT8, F6, AF8, AF4, F2.

Finally, cluster‐based permutation testing of the 88%‐condition (stimulus) versus the 50%‐condition (stimulus) indicated an effect of condition, showing two significant clusters (Figure 3c). The range of the first cluster (*p* = .013) was around 30–170 ms and included electrodes: Fp1, Fz, F3, F7, FC5, FC1, C3, T7, CP5, CP1, Pz, P3, P7, O1, O2, P4, P8, CP6, CP2, Cz, C4, FC2, AF7, AF3, AFz, F1, F5, FT7, FC3, C1, C5, TP7, CP3, P1, P5, PO7, PO3, POz, PO4, PO8, P6, P2, CPz, CP4, TP8, C6, C2. The range of the second cluster (*p* < .001) was around 180–410 ms and included electrodes: Fp1, Fz, F3, F7, FC5, FC1, C3, T7, CP5, CP1, Pz, P3, P7, O1, O2, P4, P8, CP6, CP2, Cz, C4, T8, FC6, FC2, F4, F8, Fp2, AF7, AF3, AFz, F1, F5, FT7, FC3, C1, C5, TP7, CP3, P1, P5, PO7, PO3, POz, PO4, PO8, P6, P2, CPz, CP4, TP8, C6, C2, FC4, FT8, F6, AF8, AF4, F2.

### 3.3 Omission PCA

After we established that the ERPs of the different experimental conditions differed significantly, we used PCA for signal decomposition to identify the components which carried the crucial information. PCA of the omission ERPs extracted a total of 16 components (as determined by Horn's parallel test) explaining 96.7% of variance. As no prior information was available regarding PCA separation of somatosensory omission components, selection of relevant components was based on visual inspection. Relevant components were selected based on localized peaks in the topographies of either the 88%‐condition or the 50%‐condition (although no omission components were observed in the 50%‐condition). Components were named analogous to auditory omission studies, that is, based on latency and polarity. Results of this process are summarized in Table 1 in chronological order.

**TABLE 1:** Results of PCA in chronological order. Displayed are the name of the component, the component number in the PCA, the explained variance of the PCA component, peak latency of the PCA component, and PCA component topography.

Component name	Component number	Explained variance	Peak latency	Activation topography
Early oN1	1	11.7%	90 ms	Right centrotemporal
Late oN1	10	5.9%	138 ms	Right centrotemporal
oN2	8	7.2%	172 ms	Frontal
oN3	6	7.6%	214 ms	Posterior‐temporal
oP3‐1	5	8.5%	304 ms	Fronto‐central
oP3‐2	3	9.0%	348 ms	Central
oP3‐3	4	8.7%	394 ms	Fronto‐central
oP3‐4	13	2.8%	430 ms	Right centrotemporal
oP3‐5	2	10.8%	466 ms	Right centrotemporal

Abbreviation: PCA, principal component analysis.

#### 3.3.1 Early oN1

**FIGURE 4:**
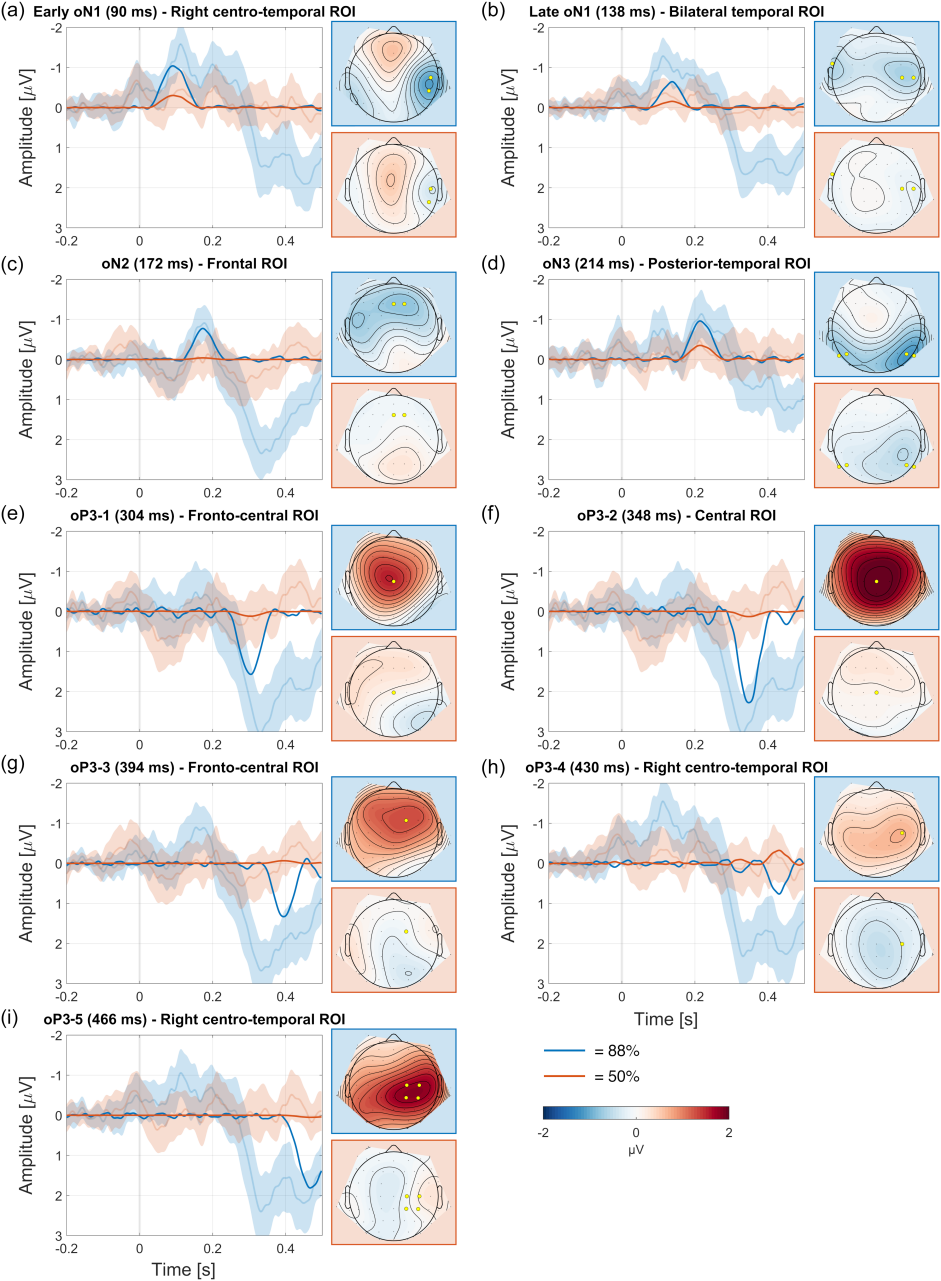
Principal component analysis (PCA) omission components in chronological order (a–i). Plots show difference waves (condition minus motor) for reconstructed PCA (opaque) and the original event‐related potentials (ERPs) including 95% CIs (transparent) at highlighted (yellow) electrodes.

PCA extracted two separate components from the first negative wave in the omission ERP. These were termed early and late oN1 (Figure 4a,b), analogous to auditory findings, where o stands for omission and N for the polarity (negative). The observed data provided decisive evidence for elicitation of the component in the 88%‐condition (*BF*_10_ = 156, *d* = 0.79, *t*(29) = 4.300, *p* < .001). In contrast, data provided weak evidence against elicitation of the component in the 50%‐condition (*BF*_10_ = 0.491, *d* = 0.26, *t*(29) = 1.438, *p* = .161). Finally, data provided moderate evidence in favour of a difference between 88%‐ and 50%‐conditions (*BF*_10_ = 8.54, *d* = 0.56, *t*(29) = 3.061, *p* = .005).

#### 3.3.2 Late oN1

The observed data provided decisive evidence for elicitation of the component in the 88%‐condition (*BF*_10_ = 93, *d* = 0.75, *t*(29) = 4.089, *p* < .001). In contrast, data provided weak evidence against elicitation of the component in the 50%‐condition (*BF*_10_ = 0.547, *d* = 0.28, *t*(29) = 1.521, *p* = .139). Finally, data provided strong evidence in favour of a difference between 88%‐ and 50%‐conditions (*BF*_10_ = 15.0, *d* = 0.61, *t*(29) = 3.316, *p* = .002).

#### 3.3.3 oN2

Analogous to auditory findings, a frontal negativity was observed around 170 ms (Figure 4c). For this reason, the same naming was applied. The observed data provided decisive evidence for elicitation of the component in the 88%‐condition (*BF*_10_ = 313, *d* = 0.84, *t*(29) = 4.580, *p* < .001). In contrast, data provided moderate evidence against elicitation of the component in the 50%‐condition (*BF*_10_ = 0.199, *d* = 0.043, *t*(29) = 0.235, *p* = .816). Finally, data provided moderate evidence in favour of a difference between 88%‐ and 50%‐conditions (*BF*_10_ = 7.585, *d* = 0.55, *t*(29) = 3.005, *p* = .005).

#### 3.3.4 oN3

Contrary to earlier findings in auditory modality (e.g., Dercksen et al., 2020; Korka et al., 2020), PCA extracted another negativity which we termed omission N3 (oN3; Figure 4d). The observed data provided decisive evidence for elicitation of the component in the 88%‐condition (*BF*_10_ = 40, *d* = 0.68, *t*(29) = 3.743, *p* < .001). In contrast, data provided weak evidence against elicitation of the component in the 50%‐condition (*BF*_10_ = 0.560, *d* = 0.28, *t*(29) = 1.539, *p* = .135). Finally, data provided moderate evidence in favour of a difference between 88%‐ and 50%‐conditions (*BF*_10_ = 7.900, *d* = 0.55, *t*(29) = 3.024, *p* = .005).

#### 3.3.5 oP3‐1

The negative polarity components were followed by a positivity (oP3), which PCA separated in five components that we termed oP3‐1 to oP3‐5 (Figure 4e–i). This naming convention was adapted from Dercksen et al. (2020), where PCA also separated the oP3 in different subcomponents. The observed data provided decisive evidence for elicitation of the component in the 88%‐condition (*BF*_10_ = 54, *d* = 0.71, *t*(29) = 3.867, *p* < .001). In contrast, data provided moderate evidence against elicitation of the component in the 50%‐condition (*BF*_10_ = 0.216, *d* = 0.09, *t*(29) = 0.473, *p* = .640). Finally, data provided decisive evidence in favour of a difference between 88%‐ and 50%‐conditions (*BF*_10_ = 58, *d* = 0.71, *t*(29) = 3.892, *p* < .001).

#### 3.3.6 oP3‐2

The observed data provided decisive evidence for elicitation of the component in the 88%‐condition (*BF*_10_ = 8394, *d* = 1.07, *t*(29) = 5.877, *p* < .001). In contrast, data provided moderate evidence against elicitation of the component in the 50%‐condition (*BF*_10_ = 0.225, *d* = 0.10, *t*(29) = 0.566, *p* = .575). Finally, data provided decisive evidence in favour of a difference between 88%‐ and 50%‐conditions (*BF*_10_ = 1596, *d* = 0.95, *t*(29) = 5.225, *p* < .001).

#### 3.3.7 oP3‐3

The observed data provided decisive evidence for elicitation of the component in the 88%‐condition (*BF*_10_ = 471, *d* = 0.87, *t*(29) = 4.743, *p* < .001). In contrast, data provided moderate evidence against elicitation of the component in the 50%‐condition (*BF*_10_ = 0.205, *d* = 0.06, *t*(29) = 0.340, *p* = .736). Finally, data provided decisive evidence in favour of a difference between 88%‐ and 50%‐conditions (*BF*_10_ = 228, *d* = 0.81, *t*(29) = 4.454, *p* < .001).

#### 3.3.8 oP3‐4

The observed data provided decisive evidence for elicitation of the component in the 88%‐condition (*BF*_10_ = 58, *d* = 0.71, *t*(29) = 3.892, *p* < .001). In contrast, data provided weak evidence against elicitation of the component in the 50%‐condition (*BF*_10_ = 0.760, *d* = 0.32, *t*(29) = 1.757, *p* = .09). Finally, data provided decisive evidence in favour of a difference between 88%‐ and 50%‐conditions (*BF*_10_ = 2563, *d* = 1.00, *t*(29) = 5.411, *p* < .001).

#### 3.3.9 oP3‐5

The observed data provided decisive evidence for elicitation of the component in the 88%‐condition (*BF*_10_ = 13,411, *d* = 1.11, *t*(29) = 6.062, *p* < .001). In contrast, data provided moderate evidence against elicitation of the component in the 50%‐condition (*BF*_10_ = 0.200, *d* = 0.05, *t*(29) = 0.250, *p* = .804). Finally, data provided decisive evidence in favour of a difference between 88%‐ and 50%‐conditions (*BF*_10_ = 1537, *d* = 0.95, *t*(29) = 5.210, *p* < .001).

### 3.4 Somatosensory PCA

A second PCA analysed the ERPs of the somatosensory evoked components. The somatosensory PCA extracted 15 components (as determined by Horn's parallel test) explaining 97.0% of variance. Of interest to the current study was the comparison of the chronologically first stimulus‐evoked components with the chronologically first omission components. Therefore, relevant stimulus‐evoked components were those that occurred from the start of the trial until, and including, the elicitation of the first omission responses (early and late oN1 at, respectively, 90 and 138 ms) in the omission PCA. This narrowed down the analysis to three stimulus‐evoked components ranging from 42 to 130 ms.

**FIGURE 5:**
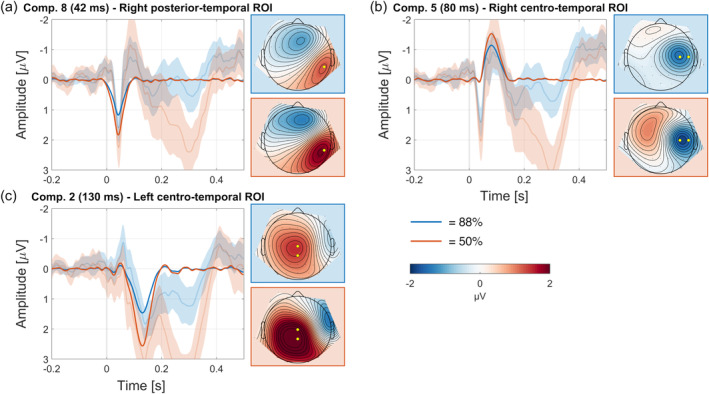
First stimulus‐evoked components in chronological order (a–c). Plots show difference wave (condition minus motor) for reconstructed principal component analysis (PCA) (opaque) and the original event‐related potentials (ERPs) including 95% CIs (transparent) at highlighted (yellow) electrodes.

Component 8 was the temporally first elicited component at 42 ms, explaining 5.0% of variance, showing a dipolar topography over right somatosensory areas (Figure 5a). The observed data provided decisive evidence for elicitation of the component in both 88%‐ and 50%‐conditions (88%‐condition: *BF*_10_ = 1830, *d* = 0.96, *t*(29) = 5.279, *p* < .001; 50%‐condition: *BF*_10_ = 8013, *d* = 1.070, *t*(29) = 5.859, *p* < .001). Data provided strong evidence for attenuation in 88%‐condition compared to 50%‐condition (*BF*_10_ = 19.3, *d* = 0.63, *t*(29) = 3.427, *p* = .002).

Component 5 was the second elicited component at 80 ms, explaining 9.6% of variance, showing a negativity over right somatosensory areas (Figure 5b). The observed data provided decisive evidence for elicitation of the component in both 88%‐ and 50%‐conditions (88%‐condition: *BF*_10_ = 75.5, *d* = 0.73, *t*(29) = 4.004, *p* < .001; 50%‐condition: *BF*_10_ = 52.6, *d* = 0.70, *t*(29) = 3.854, *p* < .001). Data provided weak evidence for attenuation in 88%‐condition compared to 50%‐condition (*BF*_10_ = 1.121, *d* = 0.37, *t*(29) = 2.006, *p* = .054).

Component 2 was the third elicited component at 130 ms, explaining 13.7% of variance, showing a dipolar topography over right somatosensory areas (Figure 5c). The observed data provided decisive evidence for elicitation of the component in both 88%‐ and 50%‐conditions (88%‐condition: *BF*_10_ = 2008, *d* = 0.97, *t*(29) = 5.315, *p* < .001; 50%‐condition: *BF*_10_ = 656, *d* = 0.89, *t*(29) = 4.875, *p* < .001). Data provided strong evidence for attenuation in 88%‐condition compared to 50%‐condition (*BF*_10_ = 11.6, *d* = 0.59, *t*(29) = 3.202, *p* = .003).

## 4 DISCUSSION

The current study tested whether an omission response would be elicited if an action‐related somatosensory prediction was violated by unexpected stimulus omission. To this end, tactile stimulation was either reliably (88%‐condition) or unreliably (50%‐condition) coupled to a self‐paced button press. Stimulus omission elicited a response in the 88%‐condition but not in the 50%‐condition. Cluster‐based permutation tests show an omission response in the 88%‐condition starting with a cluster around 80 ms that shows negative polarity in the ERP which is followed by a cluster that shows a positive ERP polarity. Temporal PCA shows a first omission component peaking at 90 ms and reveals several subcomponents within the broad negative–positive distribution of the omission ERP. We will discuss our findings in the context of somatosensory prediction, action‐effect couplings, and in comparison with studies reporting auditory omission responses.

Similar to auditory studies, a negativity around 80–100 ms is the first response to omission in the current study (Dercksen et al., 2020, 2022; Korka et al., 2020; SanMiguel, Saupe, & Schröger, 2013; SanMiguel, Widmann, et al., 2013; Stekelenburg & Vroomen, 2015; van Laarhoven et al., 2017). PCA separates the negative peak into an early (90 ms) and late (138 ms) component (Figure 4a,b). Similar results were observed in auditory studies, but whether one or two components are extracted might be dependent on the morphology of the ERP and the rotation method used for PCA (Dercksen et al., 2022). The oN1 is mainly elicited on the contralateral side of (omitted) stimulation, suggesting an origin in somatosensory‐specific areas of the left hand. This is in accordance with predictive coding: a reliable coupling between action and a sensory consequence on the left hand (88%‐condition) builds a sensory prediction, which is thought to be carried by the descending motor signal to contralateral somatosensory areas either through cortical (Jo et al., 2019; Lima et al., 2016; Pazen et al., 2020; Reznik et al., 2015; Schneider & Mooney, 2018) or subcortical (Baumann et al., 2015; Kilteni & Ehrsson, 2020; Knolle et al., 2013a; Pazen et al., 2020) connections. In case of unexpected stimulus omission, comparison of prediction and actual input results in a prediction error signal first elicited in these sensory areas, which serves to correct perception and update higher‐level models. The oN1 is assumed to represent this prediction error signal, and the fact that in both auditory and somatosensory modalities the oN1 seems to be elicited in sensory areas further supports this interpretation.

**FIGURE 6:**
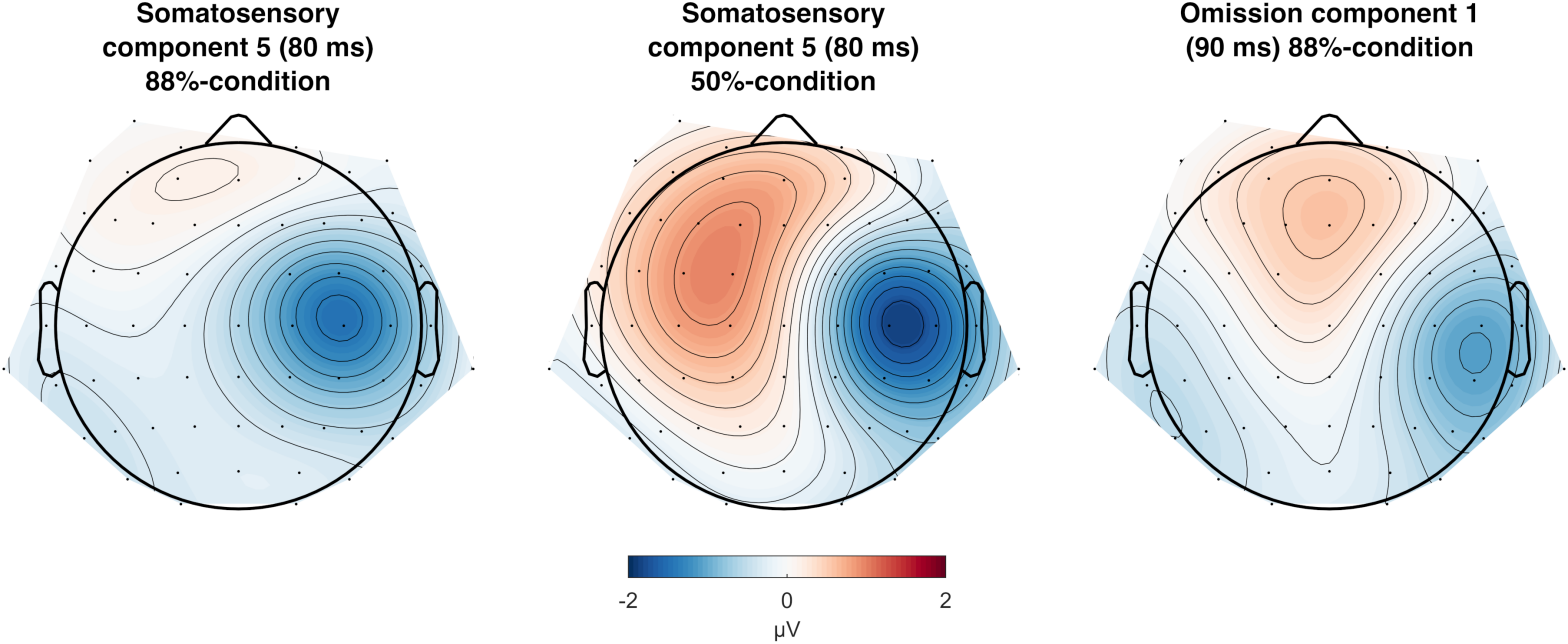
Comparison of topographies between stimulus‐evoked component 5 (88%‐condition and 50%‐condition) and omission component 1 (88%‐condition). Topographies show principal component analysis (PCA) activations at peak latency.

Cortical implementations of predictive coding assume that deeper layers of the cortical column encode prediction, while superficial layers elicit prediction error. That is, brain areas responsible for stimulus processing also generate corresponding prediction errors (Bastos et al., 2012; Jiang & Rao, 2021; Shipp, 2016). Results in the current study seem to provide some support for this hypothesis, as the early oN1 shows a similar topography and latency relative to the stimulus‐evoked component (see Figure 6 for a comparison). This component (Figure 5b), peaking around 80 ms, presumably reflects the N80 given its latency and similar topographical features compared to earlier studies (Montoya & Sitges, 2006; Schubert et al., 2008). However, the N80 is not the first cortical component that is elicited in the stimulus‐evoked potential (SEP). An earlier component around 42 ms (Figure 5a), showing topographical activation congruent with the P45 (Montoya & Sitges, 2006; Schubert et al., 2008; Van de Wassenberg et al., 2008), is elicited by tactile stimuli but does not have a counterpart in the omission response. The propagation of somatosensory predictions in this study thus seems to be limited to specific parts of the cortex. While the P45 is thought to originate from area 3b in primary somatosensory cortex (SI; Allison et al., 1992; Kakigi et al., 1995; Xiang et al., 1997), generators of the N80 have been placed in both the posterior parietal cortex (PPC) and secondary somatosensory cortex (SII; Forss, Salmelin, & Hari, 1994; Forss, Hari, et al., 1994; Forss, Jousmäki, & Hari, 1995; Hoshiyama et al., 1997). The early and late oN1 therefore seem to rather reflect activity in these latter areas, while no omission component is elicited with a latency or topography that would suggest activity from SI. Somatosensory omission results from Andersen and Dalal (2021) and Andersen and Lundqvist (2019) support this conclusion, as they observed omission responses around 135 ms with MEG showing generators localized in SII. This omission response had strong bilateral activation, which in the current study was also more prevalent in the late oN1 (138 ms). Additionally, fMRI studies demonstrate that activity in SII is attenuated when stimuli are self‐generated (Arikan et al., 2021; Blakemore et al., 1998, 2000; Kilteni & Ehrsson, 2020; Shergill et al., 2013), further supporting the notion that action‐related predictions especially influence secondary areas.

The somatosensory oN1 shows some notably similar characteristics to the auditory oN1, as the auditory oN1 resembles the topography of the t‐complex components N1a and N1c that are elicited by auditory stimuli (maximal over temporal electrode locations; Dercksen et al., 2020, 2022; SanMiguel, Saupe, & Schröger, 2013). The auditory N1a and N1c are thought to originate from the secondary auditory cortex or belt region (Bruneau et al., 1999; Näätänen & Picton, 1987; Ponton et al., 2002; Woods, 1995). As tested in these paradigms, both modalities thus show the areas adjacent to primary areas to be the main generators of prediction error in motor‐sensory couplings. This is in line with animal studies suggesting a separation of perception pathways into lemniscal areas (e.g., SI/A1), mainly propagating “raw” sensory information, and nonlemniscal areas (e.g., SII/PPC/A2), which are in comparison more sensitive to stimulus‐specific‐adaptation (SSA), prediction error generation, and deviance detection (Carbajal & Malmierca, 2018; Parras et al., 2017). The prediction error related to action‐effect couplings might therefore primarily be elicited in the rapidly responding and context‐sensitive nonlemniscal areas.

It is noteworthy that although the stimulus‐evoked P45 does not have an omission counterpart, it does demonstrate a substantial attenuation effect in the 88%‐condition. Typically, attenuated responses are interpreted as a consequence of a prediction that diminishes the elicited prediction error (Summerfield et al., 2008). This raises the question why a diminished response is observed in the P45, indicating the presence of a prediction, but no equivalent prediction error is elicited when the stimulus is omitted. The neuroscientific literature makes an important distinction here between two phenomena. On the one hand, the phenomenon referred to as local prediction (Wacongne et al., 2011), neural adaptation/refractoriness (May & Tiitinen, 2010), neural fatigue (Grill‐Spector et al., 2006), or SSA (Malmierca et al., 2014; Ulanovsky et al., 2003), which are driven by bottom‐up stimulus presentation and result in repetition suppression effects. In contrast, the phenomenon of top‐down prediction is thought to involve an influence of higher on lower cortical levels (Garrido et al., 2009), resulting in expectation suppression effects. Using appropriate paradigms, repetition suppression and expectation suppression can be disentangled, where repetition suppression presents early (around 50 ms), whereas later effects can be ascribed to expectation suppression (Todorovic & de Lange, 2012). In the 88%‐condition of the current study, the increased repetition of the tactile stimulus results in the formation of a top‐down prediction (Gijsen et al., 2021), but also in increased repetition suppression effects compared to the 50%‐condition. These latter effects are likely responsible for the attenuation observed in the P45, but are therefore not part of the top‐down prediction template associated with the motor‐somatosensory coupling. This suggests that omission responses are not merely a mirror image of sensory attenuation, but specifically reflect the prediction error related to top‐down prediction.

After the oN1 components, the next elicited omission response is the oN2 at frontal electrodes around 172 ms after button press (Figure 4c). The oN2 shows remarkable similarities in both topography and latency to the oN2 observed in auditory studies (e.g., Dercksen et al., 2020). Dercksen et al. (2020) argue that the oN2 reflects activity similar to the mismatch negativity (MMN). Extensive study of the auditory MMN has revealed separate contributing sources from temporal and frontal generators (see Deouell, 2007 for a review). The somatosensory MMN (sMMN), although not studied in as much detail, also shows evidence of both sensory‐specific and frontal generators (Kekoni et al., 1997; Kida, Nishihira, Wasaka, et al., 2004; Naeije et al., 2018; Shinozaki et al., 1998). Moreover, an intracranial study of Spackman et al. (2010) found frontal contributions to the sMMN after initial mismatch responses in somatosensory cortex. That this component is modality‐independent is further supported by results of Grundei et al. (2023), who observe similar frontal activation when comparing the auditory, somatosensory, and visual MMN. This modality‐independent, presumably preattentive component has been discussed for its role in involuntary attention switching (Spackman et al., 2010) and the processing of higher‐order prediction error (Dercksen et al., 2020; Grundei et al., 2023). The oN2 fits well to this description, as it resembles a modality‐independent frontal mismatch response that is elicited between initial sensory prediction error (oN1) and attention reorienting (oP3) responses. Additionally, this is in line with computational models that assume MMN and omission responses to be elicited by shared local circuitry (Braga & Schönwiesner, 2022).

Contrary to auditory omission studies, a third negative component was observed after the oN2, which was termed the oN3 (Figure 4d). The oN3 was elicited around 214 ms with a bilateral occipital topography that was stronger on the contralateral side of stimulation, indicating somatosensory‐specific contributions. A straightforward interpretation of this component is difficult given its absence in earlier studies and posterior topography. Studies more suited to source localisation would be helpful for understanding its possible role.

Parallel to auditory omission studies, the earlier negative components are followed by an oP3 including several subcomponents. The oP3 has been associated with the stimulus‐evoked P300 response (Dercksen et al., 2020; SanMiguel, Saupe, & Schröger, 2013; van Laarhoven et al., 2017). More specifically, the oP3‐1, oP3‐2, and oP3‐3 components demonstrate latencies and topographies that are highly congruent with the stimulus‐evoked P3a (Polich, 2007), P3b (Verleger, 2020), and novelty P3 (Barry et al., 2016). The fact that omission responses are additionally accompanied by substantial pupil responses further supports the notion that the oP3 reflects processes similar to the stimulus‐evoked P300 (Dercksen et al., 2023). The P300, which is frequently observed in somatosensory studies (Bruyant et al., 1993; Deschrijver et al., 2016; Kida, Nishihira, Hatta, et al., 2004; Schröder et al., 2021), presumably reflects higher‐order processes related to attention reorienting and knowledge updating (e.g., Barry et al., 2016; Escera et al., 1998; Polich, 2007), task demands (Schröder et al., 2021), and stimulus–response link reactivation (Verleger, 2020), and is related to the phasic activation of the locus coeruleus‐norepinephrine‐system (Nieuwenhuis et al., 2011). PCA separates the large oP3 peak in the ERP into five components (Figure 4e–i), which is a plausible result given earlier omission studies and the observed subdivision of the stimulus‐evoked P300 response into several subcomponents (e.g., Polich, 2007). What stands out in the PCA separation of the oP3 are the evident similarities of the first three components to the oP3 components observed in the auditory modality by Dercksen et al. (2020) and Korka et al. (2020), who also applied PCA. The similar elicitation of oP3 components across modalities supports that these resemble higher‐order and sensory‐unspecific cognitive processes. After the third oP3 component, two additional components were identified with similar topography that was contralateral to the stimulus hand. These components presumably reflect additional P300‐related activity. Their topographies may be compatible, for example, with neural generators in the somatosensory cortex (Tarkka et al., 1996).

The current study mainly considers observed omission responses from the perspective of motor‐somatosensory prediction. On the one hand, this is in line with the action‐based paradigm and the long history of motor‐sensory research that continues up to this day (Kilteni & Ehrsson, 2020; Korka et al., 2021; Shin et al., 2010; Sperry, 1950; von Holst & Mittelstaedt, 1950). On the other hand, research increasingly suggests that the motor system might be part of a more general prediction system. A review of Korka et al. (2021) states that both sensory and motor information likely feed into a common prediction system, where the motor system is one of multiple prediction pathways that result in similar sensory predictions. This explains the observation that similar omission responses are observed whether using motor‐sensory (Dercksen et al., 2020, Dercksen et al., 2022; Korka et al., 2020; SanMiguel, Saupe, & Schröger, 2013; SanMiguel, Widmann, et al., 2013; Stekelenburg & Vroomen, 2015) or sensory‐sensory (Stekelenburg & Vroomen, 2015; van Laarhoven et al., 2017) couplings. It also explains the similar omission response around 135 ms between the current study and studies only using rhythm to induce predictions (Andersen & Dalal, 2021; Andersen & Lundqvist, 2019). To what degree and under which circumstances the motor system plays a unique role in sensory prediction is an ongoing question. Current results suggest that an omission approach might be particularly suitable to study this question given the well‐defined subcomponents and robust activations.

## 5 CONCLUSIONS

This study investigated the prediction of tactile consequences of self‐paced actions and shows for the first time an action‐related omission response in the somatosensory modality. When a somatosensory prediction is present when pressing a button, omission of the somatosensory stimulus results in a neural response consisting of multiple consecutive components. First oN1 responses are likely elicited in secondary sensory areas. Furthermore, most of subsequent oN2 and oP3 responses are likely modality‐unspecific and presumably reflect higher order processes. The observed omission response supports the long‐standing idea that motor acts are paired with forwarded predictions of their somatosensory consequences.

## AUTHOR CONTRIBUTIONS

**Tjerk T. Dercksen**: Conceptualization, methodology, software, validation, formal analysis, investigation, data curation, writing–original draft, writing–review and editing, visualization. **Andreas Widmann**: Conceptualization, methodology, software, formal analysis, writing–review and editing. **Tömme Noesselt**: Resources, writing–review and editing. **Nicole Wetzel**: Conceptualization, methodology, resources, writing–review and editing, supervision, project administration, funding acquisition.

## FUNDING INFORMATION

This work was supported by the Center for Behavioral Brain Sciences Magdeburg financed by the European Regional Development Fund (ZS/2016/04/78120) and Leibniz Association (P58/2017). TN was funded by the Deutsche Forschungsgemeinschaft (DFG‐SFB 1436‐TPB06). Funding sources did not have any involvement in study design, data collection/analysis/interpretation, writing of the report, or decision to submit for publication.

## CONFLICT OF INTEREST STATEMENT

The authors declare no conflict of interest.

## Data Availability

The data that support the findings of this study are available from the corresponding author upon reasonable request.
